# Genomic and Functional Analyses of the Gentisate and Protocatechuate Ring-Cleavage Pathways and Related 3-Hydroxybenzoate and 4-Hydroxybenzoate Peripheral Pathways in *Burkholderia xenovorans* LB400

**DOI:** 10.1371/journal.pone.0056038

**Published:** 2013-02-13

**Authors:** María José Romero-Silva, Valentina Méndez, Loreine Agulló, Michael Seeger

**Affiliations:** Laboratorio de Microbiología Molecular y Biotecnología Ambiental, Departamento de Química and Center for Nanotechnology and Systems Biology, Universidad Técnica Federico Santa María, Valparaíso, Chile; University of Florida, United States of America

## Abstract

In this study, the gentisate and protocatechuate pathways in *Burkholderia xenovorans* LB400 were analyzed by genomic and functional approaches, and their role in 3-hydroxybenzoate (3-HBA) and 4-hydroxybenzoate (4-HBA) degradation was proposed. The LB400 genome possesses two identical *mhbRTDHI* gene clusters encoding the gentisate pathway and one *mhbM* gene encoding a 3-HBA 6-hydroxylase that converts 3-HBA into gentisate. The *pca* genes encoding the protocatechuate pathway and the *pobA* gene encoding the 4-HBA 3-monooxygenase that oxidizes 4-HBA into protocatechuate are arranged in gene clusters and single genes mainly at the minor chromosome, but also at the major chromosome and the megaplasmid. Strain LB400 was able to grow on gentisate, protocatechuate, 3-HBA and 4-HBA. Transcriptional analyses showed that the *mhbD* gene encoding the gentisate 1,2-dioxygenase was expressed during growth on 3-HBA, 4-HBA and gentisate, whereas the *pcaG* gene encoding the protocatechuate 3,4-dioxygenase was expressed only during growth on 4-HBA and protocatechuate. The *mhbM* gene encoding the 3-HBA 6-hydroxylase was transcribed in strain LB400 during growth on HBAs, gentisate, protocatechuate and glucose. The *pobA* gene encoding the 4-HBA 3-monooxygenase was expressed during growth on HBAs and glucose. 3-HBA- and 4-HBA-grown LB400 cells showed gentisate 1,2-dioxygenase activity, whereas protocatechuate 3,4-dioxygenase activity was observed only in 4-HBA-grown cells. The *mhbR* gene encoding a MarR-type transcriptional regulator that probably regulates the expression of the MhbT transporter, and the *pcaQ* and *pcaR* genes encoding LysR-type transcriptional regulators that regulate *pcaHG* and *pcaIJBDC* genes, respectively, were transcribed during growth on both HBAs, gentisate, protocatechuate and glucose, suggesting a basal constitutive expression. The results indicate active gentisate, protocatechuate, 3-HBA and 4-HBA catabolic pathways in *B. xenovorans* LB400 and suggest that 3-HBA is channeled exclusively through the gentisate route, whereas 4-HBA is funneled into the protocatechuate central pathway and potentially into the gentisate pathway.

## Introduction

Aerobic bacterial degradation of aromatic compound proceeds generally in two phases. In the first phase, the aromatic compound is prepared for ring cleavage by a variety of ring modification reactions. The second phase of aerobic degradation includes ring fission of the aromatic compound and subsequent reactions leading to the formation of Krebs cycle intermediates [1]–[4]. *Betaproteobacteria* of the *Burkholderiales* order possess an amazing metabolic versatility to degrade a large number of aromatic compounds [1], [3]. *Burkholderia xenovorans* LB400 is a bacterium able to degrade polychlorobiphenyls (PCBs) and diverse aromatic compounds [3]–[8]. LB400 genome has a size of 9.73 Mbp distributed in three replicons: the major chromosome (C1; 4.90 Mbp), the minor chromosome (C2; 3.36 Mbp) and the megaplasmid (MP; 1.47 Mbp) [3]. Genomic analysis of *B. xenovorans* strain LB400 revealed the presence of genes encoding an unusual high number of central and peripheral pathways for the degradation of aromatic compounds [3]. A recent study confirmed functionality of some predicted aromatic pathways [4]. However, the function of genes encoding diverse aromatic catabolic routes including the gentisate and protocatechuate pathways of *B. xenovorans* strain LB400 remains to be elucidated.

The gentisate pathway is a central route for the bacterial aerobic catabolism of benzoates such as 3-HBA and salicylate (2-hydroxybenzoate), phenolic compounds such as *m*-cresol and 2,5-xylenol, and polycyclic aromatic hydrocarbons including naphthalene and phenanthrene [9]–[14]. Gentisate is cleaved by gentisate 1,2-dioxygenase (MhbD) to form maleylpyruvate. Maleylpyruvate is then isomerized by a glutathione-dependent maleylpyruvate isomerase (MhbI) to yield fumarylpyruvate. A fumarylpyruvate hydrolase (MhbH) catalyzes fumarylpyruvate conversion into pyruvate and fumarate.

The gentisate catabolic pathway has been described as the central route for 3-HBA degradation in some bacteria. 3-HBA is degraded through the gentisate central pathway by the 3-HBA 6-hydroxylase in *Burkholderia cepacia* J2315 [15], *Pseudomonas alcaligenes* P25X1 [9], *Klebsiella pneumoniae* M5a1 [11], [16] and *Salmonella typhimurium* [12]. The 3-HBA 6-hydroxylase is encoded by the *mhbM* gene in *K. pneumoniae* M5a1 [11], [16]. Alternatively, 3-HBA could be degraded through the protocatechuate catabolic pathway by the 3-HBA 4-hydroxylase, which is encoded by the *mobA* gene in *Comamonas testosteroni* KH122 [17], [18].

The protocatechuate pathway is a central catabolic route for aromatic compounds, which is widely distributed among taxonomically diverse bacteria and fungi [1]–[3], [19], [20]. Protocatechuate is a key central intermediate in bacterial degradation of diverse aromatic compounds, including 4-HBA, 3-HBA, vanillate, ferulate, coniferyl alcohol, *p*-coumarate, phthalate, isophthalate and terephthalate. Protocatechuate oxygenolytic ring-cleavage is catalyzed by protocatechuate 3,4-dioxygenase (PcaGH) to generate 3-carboxy-*cis*,*cis*-muconate, which is converted into 4-carboxymuconolactone by 3-carboxy-*cis*,*cis*-muconate cycloisomerase (PcaB). 4-Carboxymuconolactone decarboxylase (PcaC) transforms the 4-carboxymuconolactone into β-ketoadipate enol-lactone, which is then hydrolyzed by β-ketoadipate enol-lactone hydrolase (PcaD) into β-ketoadipate. The enzyme β-ketoadipate succinyl-CoA tranferase (PcaIJ) converts β-ketoadipate into β-ketoadipyl-CoA, which is finally transformed into succinyl-CoA and acetyl-CoA by β-ketoadipyl-CoA thiolase (PcaF).

The protocatechuate central pathway is involved in 4-HBA degradation in some microorganisms. 4-HBA is hydroxylated by 4-HBA 3-monooxygenase encoded by the *pobA* gene to yield protocatechuate in *Pseudomonas* and *Cupriavidus* strains [2], [21], [22].

The aims of this study were to characterize the gentisate and protocatechuate central pathways in *B. xenovorans* LB400 using genomic and functional approaches. In addition, the role of these ring-cleavage pathways during 3-HBA and 4-HBA degradation in strain LB400 was studied. This report describes for the first time the metabolism of 3-HBA and 4-HBA in *B. xenovorans* LB400. This study demonstrates functional gentisate and protocatechuate catabolic pathways in *B. xenovorans* LB400 that are involved in the degradation of 3-HBA and 4-HBA.

## Materials and Methods

### Chemicals

3,4-dihydroxybenzoic acid (protocatechuate, >98% purity), 2,5-dihydroxybenzoic acid (gentisate, 98% purity), 3-hydroxybenzoic acid (>99% purity) and 4-hydroxybenzoic acid (≥99% purity) were obtained from Sigma-Aldrich (Saint Louis, MO, USA).

### Bacterial Strain and Culture Conditions

*B. xenovorans* strain LB400 was cultivated in M9 mineral medium with trace solution and glucose (5 mM) [23], 3-HBA (5 and 10 mM), 4-HBA (5 mM), gentisate (5 mM) or protocatechuate (5 mM) as sole carbon and energy source at 30°C [3], [23]. Growth was determined by measuring turbidity at 525 nm and by counting colony-forming units (CFU). Aliquots taken from bacterial cultures were diluted and plated on Luria-Bertani (LB) medium. CFU mL^−1^ values were calculated as the mean ± SD of values from at least three independent experiments.

### Isolation of RNA and RT-PCR

RNA was isolated from LB400 cells grown until mid-exponential growth phase (turbidity _525 nm_ 0.4–0.6) on 3-HBA, 4-HBA and glucose. RNA was isolated using RNeasy mini kit (Qiagen, Hilden, Germany). DNase I treatment was performed using the RNase-Free DNase Set (Qiagen, Hilden, Germany). The RNA was quantified using a Qubit fluorometer (Invitrogen, Carlsbad, CA, USA). In this study, specific primers for *mhbD* (BxeA2627, BxeA4526), *mhbT* (BxeA2625, BxeA4528), *mhbR* (BxeA2624, BxeA4529), *mhbM* (BxeB2526), *pcaG* (BxeB2776), *pcaQ* (BxeB2772), *pcaR* (BxeB0642) and *pobA* (BxeA2040) genes were designed and used (Table 1). Reverse transcription-PCR (RT-PCR) was carried out with 40 µg of total RNA and the sequence-specific primers using SuperScript One-step RT-PCR with Platinum Taq (Invitrogen, Carlsbad, CA, USA). Amplification of the 16S rRNA gene was performed as control for DNA contamination using the primers 27f (5′-AGAGTTTGATCMTGGCTCAG-3′) and 1492r (5′-TACGGYTACCTTGTTACGACTT-3′) as reported [4]. Negative and positive controls were included in each RT-PCR assay. The expression of the constitutively expressed 16S rRNA was used as a control to normalize across samples. At least three independent RNA samples were collected at each condition and two independent RT-PCR reactions for each sample were done to assess reproducibility.

**Table 1:** Primer sets designed and used in this study.

Gene	Name	Sequence (5′- 3′)	Reference
*mhbD1* *mhbD2*	mhbDf	5′-ATCCGGGGGACTTTATCATC-3′	This study
*mhbD1* *mhbD2*	mhbDr	5′-GCGATTCGAGTAGCTGAACA-3′	This study
*mhbR1* *mhbR2*	mhbRf	5′-GCAAGTTCGCCACAATTCCGTT-3′	This study
*mhbR1* *mhbR2*	mhbRr	5′-TTGCGTTACGGTAAAGGCGGAT-3′	This study
*pcaG*	pcaGf	5′-AAGACAGTTTCCTTCTCGCCCTG-3′	This study
*pcaG*	pcaGr	5′-CTCAAGCAAACGCCTTCGCAA-3′	This study
*pcaQ*	pcaQf	5′-AACTGTTCAGCGAGCCGTTGAT-3′	This study
*pcaQ*	pcaQr	5′-AAAAGGCAGCGGCAAACGTA-3′	This study
*pcaR*	pcaRf	5′-ATGCTCGACGTGCAGGAATTCA-3′	This study
*pcaR*	pcaRr	5′-TGAAACTTGCGATGCCAGTGCT-3′	This study
*mhbM*	mhbMf	5′-TGACCATTCTGGAACAGGCGAA-3′	This study
*mhbM*	mhbMr	5′-TATACGGCACATCAATCGCGCT-3′	This study
*pobA*	pobAf	5′-TTGATAGCGTGGTGCTGGAA-3′	This study
*pobA*	pobAr	5′-ACGCTATCTTTCGGATCGCA-3′	This study
16S rRNA	27F	5′-AGAGTTTGATCMTGGCTCAG-3′	[4], [23]
16S rRNA	1492R	5′-TACGGYTAC CTTGTTACGACTT-3′	[4], [23]

### Preparation of Bacterial Cell Extracts

Cells grown until exponential phase with 3-HBA, 4-HBA or glucose were harvested by centrifugation (10,733 g for 10 min) at 4°C. Bacterial cells washed with sodium phosphate buffer (50 mM, pH 7.0) and concentrated were disrupted using ultrasonic cells disruptor Microson at 4°C. Cell lysates were clarified by centrifugation (19,300 g for 15 min) at 4°C. The protease inhibitor cocktail Tablet Complete mini (Roche, Indianapolis, USA) was added to cell extracts to reach a final concentration of 0.8 mg/ml. Protein concentration was measured using the Quantit-iT Protein Assay kit (Invitrogen, Carlsbad, CA, USA) that uses a fluorescent dye whose quantum yields are enhanced significantly when binding at the detergent-protein interface and measuring fluorescence at room temperature with the Qubit fluorometer (Invitrogen, Carlsbad, CA, USA) [24].

### Dioxygenase Activity Assays

Gentisate 1,2-dioxygenase activity was determined spectrophotometrically by measuring the formation of maleylpyruvate at 330 nm [13]. The assay volume (10 ml) contained sodium phosphate buffer (50 mM, pH 7.0), crude extract (1000 µg of protein) and gentisate (0.6 mM). The reactions were carried out at 30°C and initiated by the addition of gentisate. Gentisate 1,2-dioxygenase activity was calculated using the molar extinction coefficient of maleylpyruvate 10,300 M^−1^ cm^−1^ [13], [25]. Protocatechuate 3,4-dioxygenase activity was determined spectrophotometrically by the disappearance of protocatechuate measuring the absorbance at 290 nm (A_290 nm_) [26]. The assay volume (10 ml) contained sodium phosphate buffer (50 mM, pH 7.0), crude extract (1000 µg of protein) and protocatechuate (0.6 mM). The reactions were carried out at 30°C and initiated by the addition of protocatechuate. Protocatechuate 3,4-dioxygenase activity was calculated using the difference of the molar extinction coefficient of protocatechuate and its product under these conditions that is 2,280 M^−1^ cm^−1^ [27]. One enzyme unit corresponds to the transformation under the conditions described above of 1 µmol of substrate to product per minute per milligram of protein at 30°C. The enzymatic activity assays were done in triplicate.

## Results

### Genomic Analysis of the Gentisate Pathway and Related Peripheral Routes

**Figure 1:**
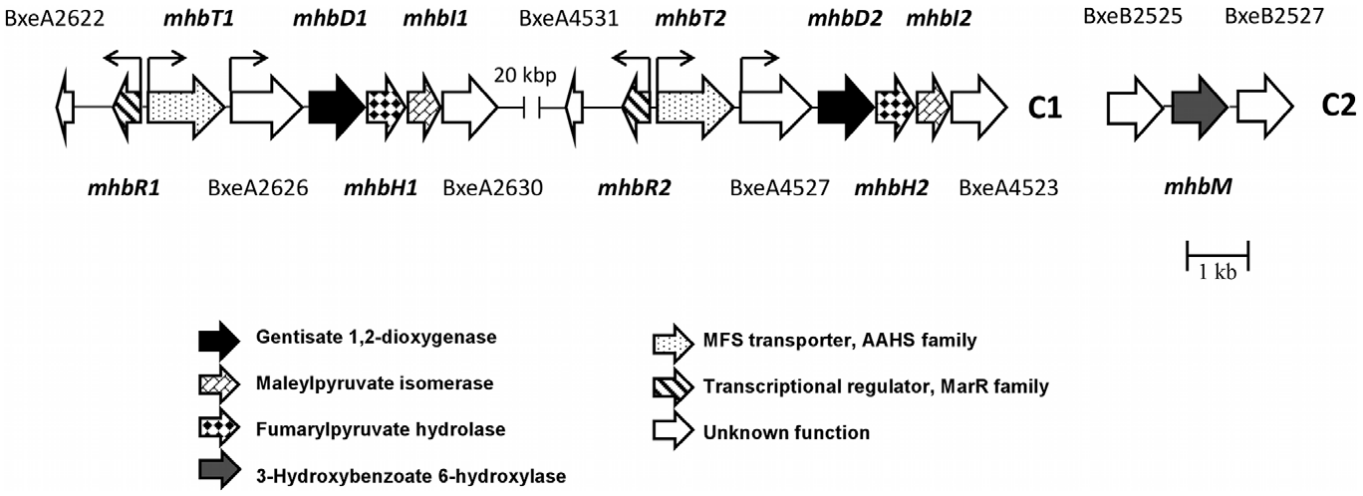
Predicted *mhb* genes encoding the gentisate central pathway and related genes in *B. xenovorans* LB400. The *mhb* genes located at the major chromosome (C1) and the minor chromosome (C2). The genes encoding gentisate 1,2-dioxygenase are indicated with black arrows. The orientations of ORFs are represented by open arrows. The promoter regions for *mhbR1*, *mhbR2, mhbT1, mhbT2,* BxeA2626 and BxeA4527 are denoted with small black arrows, bent in the directions of transcription. The sizes of the genes and the intergenic regions are on scale.

**Figure 2:**
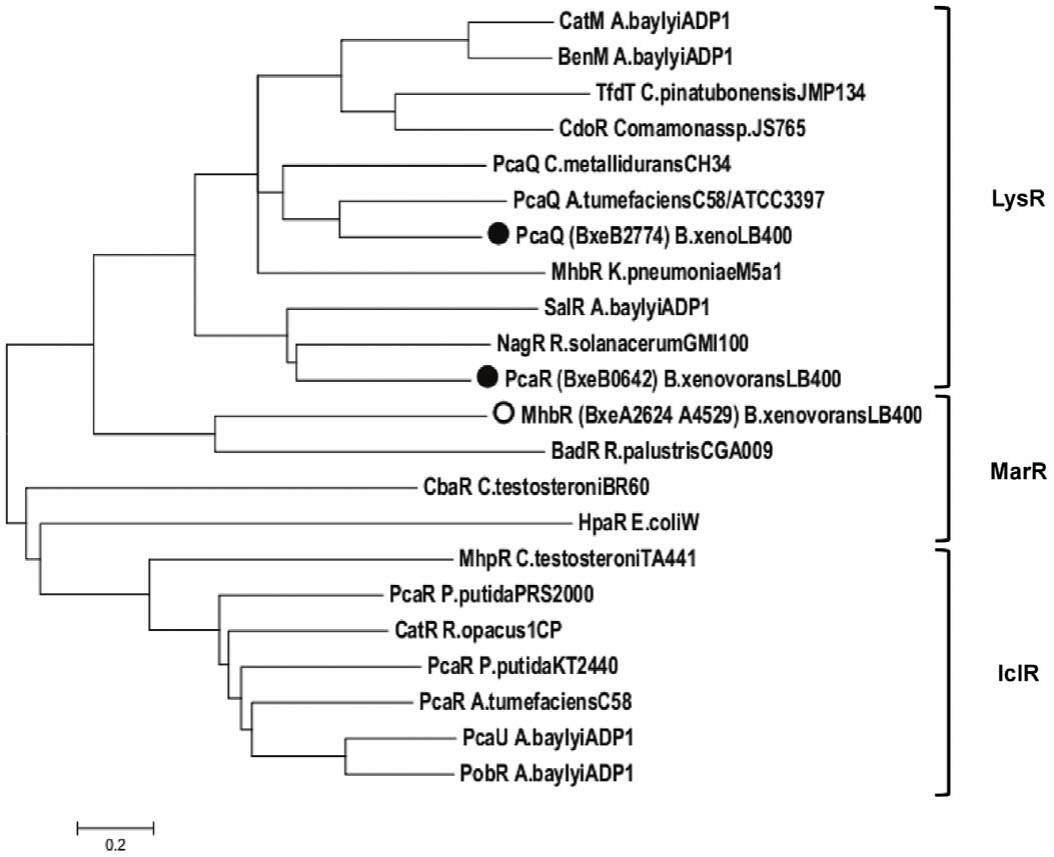
Relationship between bacterial transcriptional regulators for gentisate and protocatechuate catabolic pathways. LysR, MarR and IclR transcriptional regulators from *B. xenovorans* LB400 and other bacteria were depicted. The dendogram was obtained by the neighbor-joining method using MEGA 5.0 based on sequence alignment calculated by CLUSTAL W using the default options. Circles denote proteins encoded in the genome from *B. xenovorans* LB400. The sequence and their accession number are: CatM, *Acinetobacter baylyi* ADP1 (P07774); BenM, *A. baylyi* ADP1 (AAC46441); TfdT, *Cupriavidus pinatubonensis* JMP134 (AAC44724); CdoR, *Comamonas* sp. JS765 (AAC79916); MhbR, *Klebsiella pneumoniae* M5a1 (Q5EXK6); PcaQ, *Cupriavidus metallidurans* CH34 (ABF10579); PcaQ, *Agrobacterium tumefaciens* C58/ATCC3397 (P0A4T6); PcaQ, *B. xenovorans* LB400 (Q13RR8); SalR, *A. baylyi* ADP1 (AAF04311); NagR, *Ralstonia* sp. U2 (Q9EXL7); PcaR, *B. xenovorans* LB400 (ABE35311); MhbR, *B. xenovorans* LB400 (ABE30329); BadR, *Rhodopseudomonas palustris* CGA009 (AAC23923); CbaR, *Comamonas testosteroni* TA441 (AAG00065); HpaR, *Escherichia coli* W (Z37980); MhpR, *C. testosteroni* TA441 (P77569); PcaR, *Pseudomonas putida* PRS2000 (Q52154); CatR, *Rhodococcus opacus* (Q33539); PcaR, *P. putida* KT2440 (Q88N41); PcaR, *A. tumefaciens* C58 (Q7CV82); PcaU, *A. baylyi* ADP1 (AAC37157); PobR, *A. baylyi* ADP1 (A36893).

The *mhb* genes encoding the gentisate catabolic pathway were analyzed in the genome of *B. xenovorans* LB400. Table 2 describes the predicted genes encoding the gentisate central pathway and related peripheral routes in *B. xenovorans* LB400. Two identical *mhbRTDHI* gene clusters encoding the gentisate pathway were identified at C1 (Fig. 1). Both *mhb* gene clusters are located adjacent in two identical chromosomal regions comprising thirty genes. Sequence analyses suggest that *mhbD, mhbH* and *mhbI* genes of strain LB400 encode the gentisate 1,2-dioxygenase, maleylpyruvate isomerase and fumarylpyruvate hydratase, respectively. The *mhbD, mhbH* and *mhbI* gene products of strain LB400 possess >52% sequence identity with the respective MhbD, MhbH and MhbI proteins from *K. pneumoniae* M5a1 [13], [30]. Sequence analyses of both *mhbDHI* gene clusters neighborhoods in strain LB400, revealed that the BxeA2625 and BxeA4528 (hereafter, *mhbT1* and *mhbT2*) genes encode major facilitator superfamily (MFS) transporters that belong to the aromatic acid:H^+^ symporter (AAHS) family. Genomic context suggests that the *mhbT1* and *mhbT2* genes encode a MFS transporters probably involved in gentisate transport in *B. xenovorans* LB400. The BxeA2624 and BxeA4529 genes (hereafter, *mhbR1* and *mhbR2*) were located upstream and adjacent to the *mhbT* genes, and divergently transcribed. The *mhbR* gene product shares 30% sequence identity with MarR-type transcriptional regulator related to the *mhb* gene cluster from *Polaromonas naphthalenivorans* CJ2 (Table 2). Sequence alignment of transcriptional regulators associated to central aromatic pathways showed that the *mhbR* gene product from *B. xenovorans* LB400 is a MarR-type transcriptional regulator (Fig. 2), which probably regulates the expression of the *mhbT* gene. The MhbR regulator in *K. pneumoniae* M5a1 is a LysR-type activator of the *mhbTDHI* gene cluster [39]. Genome analyses indicated that two divergent σ^70^-type promoters were present between *mhbR* and *mhbT*. A regulatory binding site with a palindromic sequence for a MarR-type transcriptional regulator (5′-ATTGTTTCACAAACTAAT-3′) was identified upstream from the *mhbT* gene promoter, which is similar to the consensus MarR-type regulator binding site (5′-(T/A)(A/T)(T/A)CTT-N_6_-(A/G)A(G/A)(A/T)(T/AC)(A/T) -3′) [40].

**Table 2:** Predicted genes encoding the gentisate and protocatechuate pathways and peripheral reactions from *B. xenovorans* LB400.

Gene*	ORF	aa	Related gene products			
			Protein	Function	Organism (Reference)	% Id (aa)
*mhbR1 mhbR2*	BxeA2624 BxeA4529	166	MhbR	Transcriptional regulator, MarR family	*Polaromonas naphthalenivorans CJ2* [28]	30 (161)
*mhbT1* *mhbT2*	BxeA2625BxeA4528	466	PcaK	4-Hydroxybenzoate transporter	*Pseudomonas putida* PRS2000 [29]	33 (448)
*mhbD1* *mhbD2*	BxeA2627BxeA4526	348	MhbD	Gentisate-1,2-dioxygenase	*Klebsiella pneumoniae* M5a1 [30]	64 (345)
*mhbH1* *mhbH2*	BxeA2628BxeA4525	232	MhbH	Fumarylpyruvate hydrolase	*K. pneumoniae* M5a1 [30]	63 (232)
*mhbI1mhbI2*	BxeA2629BxeA4524	216	MhbI	Maleylpyruvate isomerase	*K. pneumoniae* M5a1 [30]	53 (214)
*mhbM*	BxeB2526	396	MhbM	3-Hydroxybenzoate 6- hydroxylase	*K. pneumoniae* M5a1 [30]	38(397)
*pcaG*	BxeB2776	195	PcaG	Protocatechuate 3,4- dioxygenase, alpha subunit	*Acinetobacter lwoffii* K24 [31]	90 (132)
*pcaH*	BxeB2775	234	PcaH	Protocatechuate 3,4- dioxygenase, beta subunit	*A. lwoffii* K24 [31]	96 (226)
*pcaQ*	BxeB2772	310	PcaQ	Transcriptional regulator, LysR family	*Agrobacterium tumefaciens* A348 [32]	48 (311)
*pcaF1*	BxeB2167	400	PcaF	β-Ketoadipyl-CoA thiolase	*P. putida* PRS2000 [33]	71 (400)
*pcaF2*	BxeA0469	400	PcaF	β-Ketoadipyl- CoA thiolase	*P. putida* PRS2000 [33]	69 (400)
*pcaF3*	BxeA4255	402	PcaF	β-Ketoadipyl-CoA thiolase	*P. putida* PRS2000 [33]	42 (400)
*pcaC*	BxeB0647	130	PcaC	4-Carboxymucolactone decarboxylase	*Acinetobacter baylyi* ADP1 [34]	54 (134)
*pcaD1*	BxeB0646	263	PcaD	β-Ketoadipate enol-lactone hydrolase	*A. baylyi* ADP1 [35]	38 (266)
*pcaD2*	BxeB0595	265	PcaD	β-Ketoadipate enol-lactone hydrolase	*A. baylyi* ADP1 [35]	31 (266)
*pcaB1*	BxeB0645	459	PcaB	3-Carboxy*-cis,cis*-muconate cycloisomerase	*P. putida* PRS2000 [36]	52 (422)
*pcaB2*	BxeB1906	445	PcaB	3-Carboxy*-cis,cis*-muconate cycloisomerase	*P. putida* PRS2000 [36]	34 (422)
*pcaJ1*	BxeB0644	219	PcaJ	β-Ketoadipate:succinyl-CoA transferase, beta subunit	*P. putida* PRS2000 [37]	77 (213)
*pcaJ2*	BxeC0574	265	PcaJ	β-Ketoadipate:succinyl-CoA transferase, beta subunit	*P. putida* PRS2000 [37]	53 (213)
*pcaJ3*	BxeA1366	234	PcaJ	β-Ketoadipate:succinyl-CoA transferase, beta subunit	*P. putida* PRS2000 [37]	44 (213)
*pcaI1*	BxeB0643	233	PcaI	β-Ketoadipate:succinyl-CoA transferase, alpha subunit	*P. putida* PRS2000 [37]	79 (231)
*pcaI2*	BxeC0572	235	PcaI	β-Ketoadipate:succinyl-CoA transferase, alpha subunit	*P. putida* PRS2000 [37]	49 (231)
*pcaR*	BxeB0642	309	PcaR	Transcriptional regulator, LysR family	*Burkhoderia phytofirmans* PsJN [38]	95 (309)
*pobA*	BxeA2040	394	PobA	4-Hydroxybenzoate-3-monooxygenase	*P. fluorescens* Pf-5 [21]	51 (394)

* Genes for those cases where biochemical or genetic evidence for function is available are underlined.

Genome analysis indicated that an independent σ^70^-type promoter is located upstream of the BxeA2626 (BxeA4527) and the *mhbDHI* genes. Upstream from the BxeA2626 (BxeA4527) gene promoter is located a LysR-type regulator binding sequence (5′-ATTCACTTCGAGAAT-3′) that has the conserved (5′-T-N_11_-A-3′) motif critical for binding of LysR-type transcriptional regulators [41]. The specific LysR-type transcriptional regulator gene of the *mhbDHI* gene cluster of strain LB400 has not been identified in this study.

**Figure 3:**
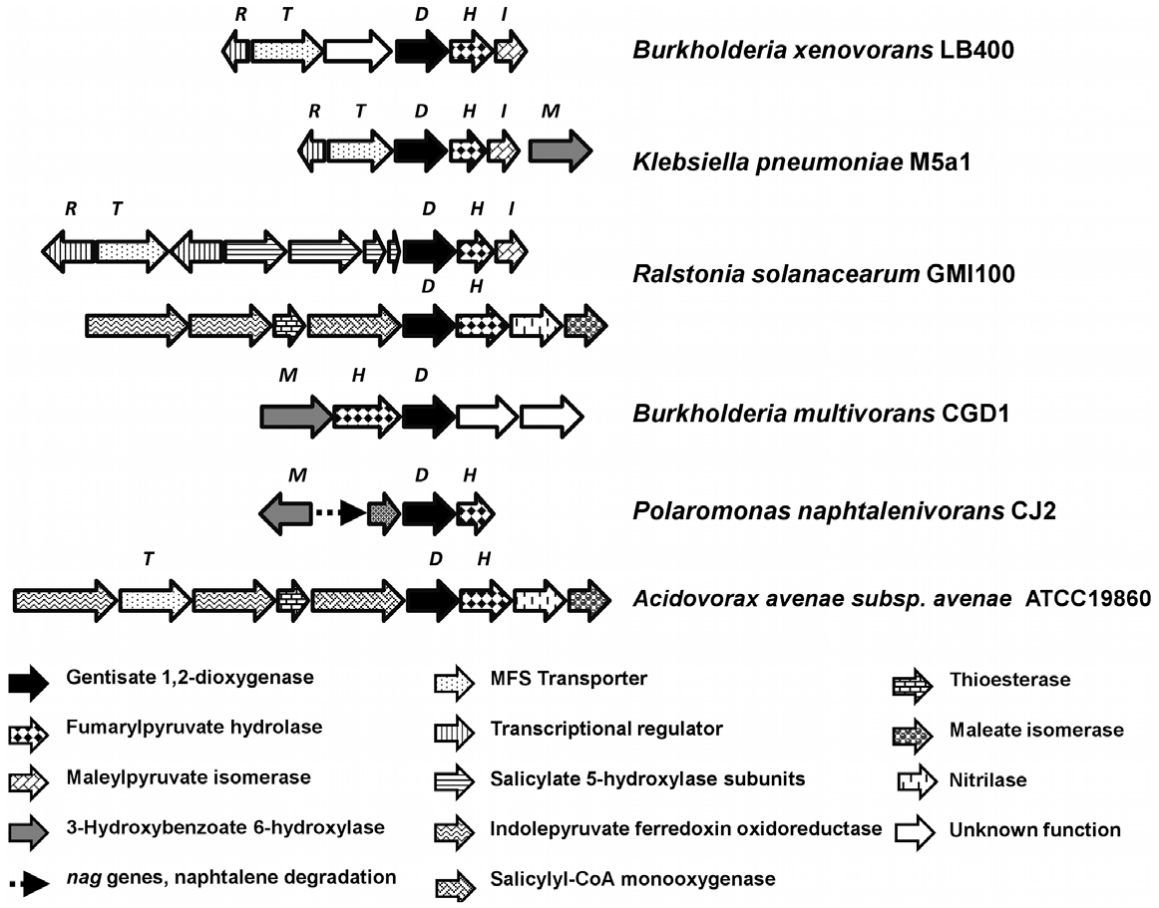
Organization of the gene clusters involved in the catabolism of gentisate in *B. xenovorans* LB400 and other bacteria. The genes encoding gentisate 1,2-dioxygenase are indicated with black arrows. The sizes of the genes and the intergenic regions are on scale.

Gene organization of the *mhb* cluster in *B. xenovorans* LB400 is similar to the *mhb* cluster from *K. pneumoniae* M5a1, except for the presence of a coding sequence of unknown function between *mhbT* and *mhbD* genes in strain LB400, the presence of the *mhbM* gene encoding 3-HBA 6-hydroxylase located downstream to *mhbI* in strain M5a1 and that the *mhbR* genes encode different transcriptional regulators (Fig. 3). The *mhb* gene clusters from other *Burkholderiales* strains including *Ralstonia solanacearum* GMI100, *B. multivorans* CGD1, *P. naphthalenivorans* CJ2 and *Acidovorax avenae* subsp. avenae ATCC19860 showed different *mhb* gene organizations (Fig. 3).

A search of the gene encoding a 3-HBA 6-hydroxylase that funnel 3-HBA into the gentisate central pathway was performed in the LB400 genome. Sequence analysis indicates that the BxeB2526 gene (hereafter *mhbM*) located at C2 encodes a 3-HBA 6-hydroxylase (Fig. 1). The *mhbM* gene product from strain LB400 shared a 38% sequence identity with 3-HBA 6-hydroxylase (MhbM) from *K. pneumoniae* M5a1 [30]. A 4-HBA 1-hydroxylase activity that converts 4-HBA into gentisate has been described [42]. However, the gene sequence encoding 4-HBA 1-hydroxylase has not been reported yet, impeding a further genomic analysis.

### Genomic Analysis of Protocatechuate Pathway and Related Peripheral Routes

**Figure 4:**
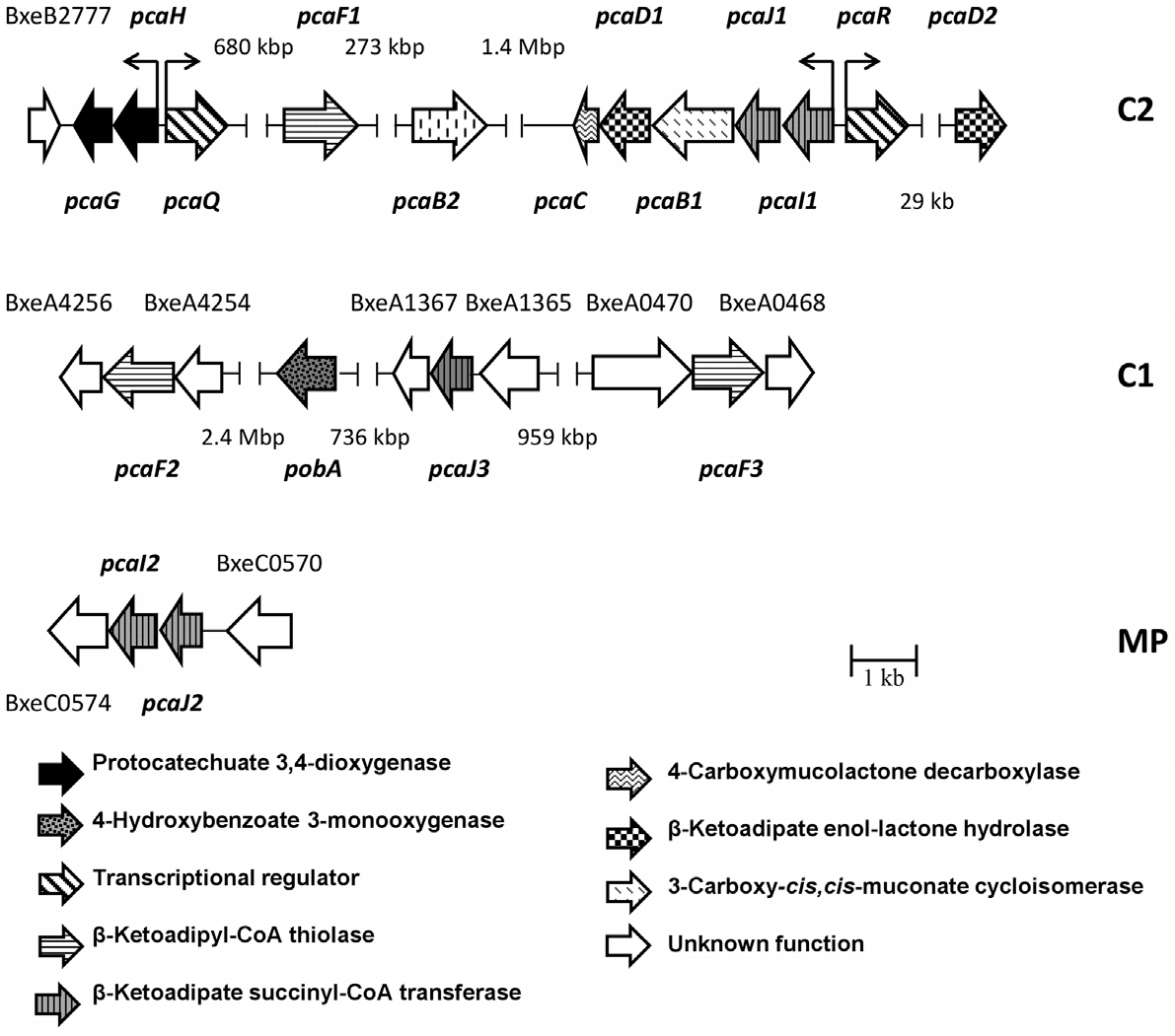
Genetic organization of the *pca* genes involved in protocatechuate catabolism in *Burkholderia xenovorans* LB400. The *pca* genes are located in the minor chromosome (C2), major chromosome (C1) and megaplasmid (MP). The genes encoding the alpha and beta subunits of the protocatechuate 3,4-dioxygenase are indicated with black arrows. The orientations of ORFs are represented by open arrows. The promoter regions for *pcaH*, *pcaQ, pcaI* and *pcaR* are denoted with small black arrows, bent in the directions of transcription. The sizes of the genes and the intergenic regions are on scale.

**Figure 5:**
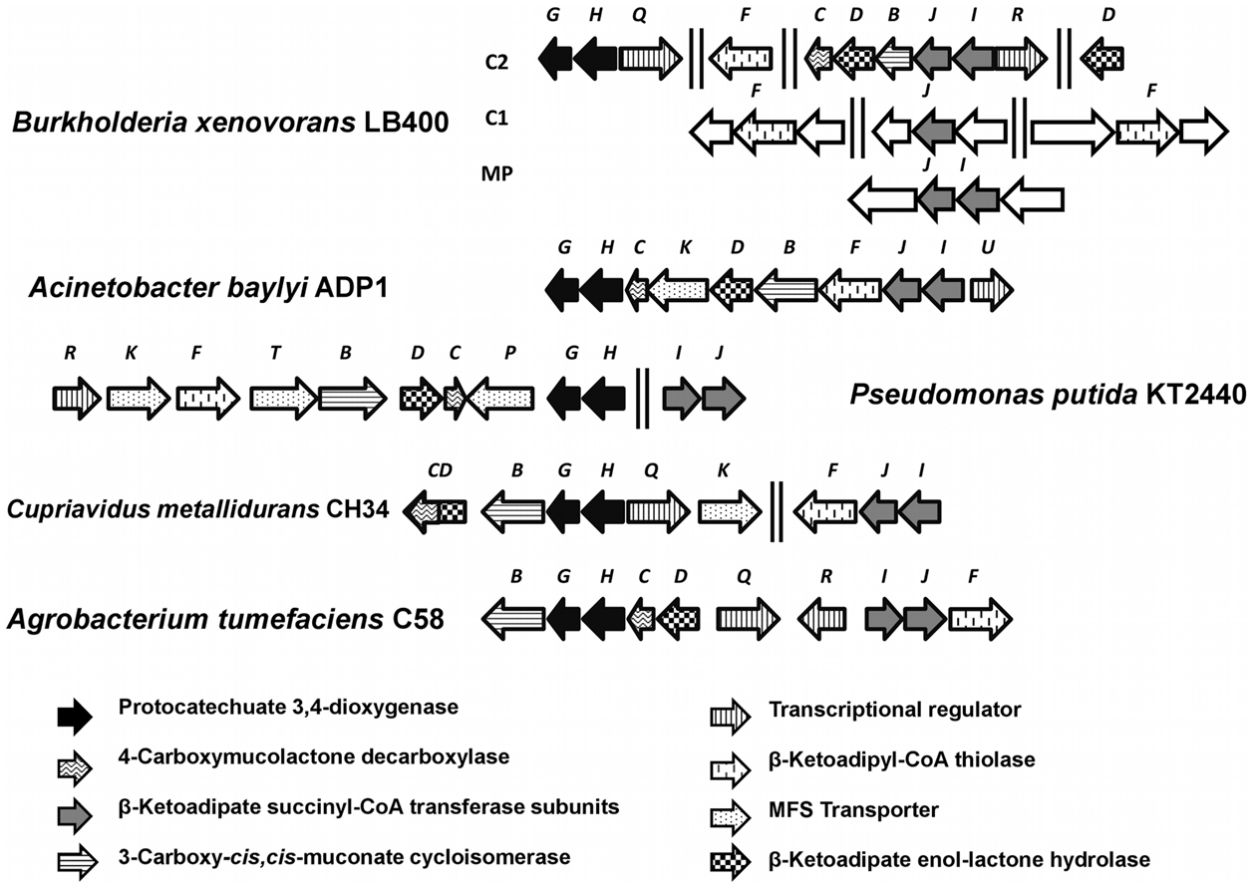
Organization of the *pca* genes involved in the catabolism of protocatechuate in *B. xenovorans* LB400 and other bacteria. The genes encoding the alpha and beta subunits of the protocatechuate 3,4-dioxygenase are indicated with black arrows. The sizes of the genes and the intergenic regions are on scale.

Predicted *pca* genes encoding the protocatechuate central pathway are distributed within the *B. xenovorans* LB400 genome (Fig. 4). Predicted *pca* genes encoding the protocatechuate pathway and related peripheral routes are listed in Table 2. Sequence analyses suggest that *pcaG*, *pcaH*, *pcaQ*, *pcaF* (BxeB2167; hereafter *pcaF1*), *pcaC*, *pcaD* (BxeB0646; hereafter *pcaD1*), *pcaB* (BxeB0645; hereafter *pcaB1*), *pcaJ* (BxeB0644; hereafter *pcaJ1*), *pcaI* (BxeB0643; hereafter *pcaI1*), *pcaQ* and *pcaR* genes encoding the protocatechuate catabolic pathway are located at C2. Copies of *pcaB* (BxeB1906; hereafter *pcaB2*) and *pcaD* genes (BxeB0595; hereafter *pcaD2*) were identified at C2. Two *pcaF* gene copies (hereafter *pcaF2* and *pcaF3,* respectively), and a *pcaJ* gene copy (hereafter *pcaJ3*) were identified by sequence analyses at C1. In addition, *pcaJ* and *pcaI* genes copies (hereafter *pcaJ2* and *pcaI2,* respectively) were identified by sequence analyses at MP. The *pcaG* and *pcaH* genes encoding the alpha and beta subunits from a protocatechuate 3,4-dioxygenase were located 2.3 Mbp distant region from *pcaIJBDC* gene cluster. The *pcaG* and *pcaH* gene products of strain LB400 showed ≥90% identity with the PcaG and PcaH proteins from *Acinetobacter lwoffii* K24 [31]. The spread in the organization of *pca* genes and genes encoding related peripheral pathways in *B. xenovorans* LB400 is also observed in *Cupriadus metallidurans* CH34 (Fig. 5). Predicted *pcaQ* gene located next to the *pcaHG* genes and divergently transcribed encodes a LysR-type transcriptional regulator (Fig. 2) that might regulate the expression of the *pcaHG* gene cluster. The PcaQ protein shared a 48% sequence identity with the LysR-type transcriptional regulator PcaQ from *Agrobacterium tumefaciens* A348 (Table 2) [43]. Two divergent σ^70^-type promoters are present between the *pcaQ* gene and the *pcaGH* gene cluster. A PcaQ LysR-type regulatory binding sequence (5′-ATAACGGCAGGTTAT-3′) was identified upstream of the *pcaH* gene promoter. This regulatory sequence has the consensus sequence for the PcaQ regulator recognition site sequence of *Burkholderiales* (5′-ATAACN_5_GTTAT-3′) [43]. Predicted *pcaR* gene (BxeB0642) located adjacent to the *pcaIJBDC* genes and divergently transcribed encodes a LysR-type transcriptional regulator (Fig. 2). The *pcaR* gene product shared a 95% sequence identity with a LysR-type transcriptional regulator adjacently located to the *pcaIJBDC* gene cluster from *B. phytofirmans* PsJN (Table 2) [38]. Genomic analysis of the *pcaCD1B1J1I1R* gene cluster showed that two divergent σ^70^-type promoters are present between the *pcaR* gene and the *pcaCD1B1J1I1* gene cluster. A putative LysR-type regulatory binding sequence (5′-GTTCGCTAAACGAAT-3′) was identified upstream to the *pcaR* gene promoter. This sequence has the conserved (5′-T-N_11_-A-3′) motif critical for binding of LysR-type autoregulatory transcriptional regulators [41].

The genes encoding the enzymes of the 3-HBA and 4-HBA peripheral reactions that funnel into the protocatechuate pathway were searched in the LB400 genome. The *pobA* gene (BxeA2040) encoding for a 4-HBA 3-monooxygenase that converts 4-HBA into protocatechuate was identified by sequence analyses at C1 (Fig. 4). The *pobA* gene protein from strain LB400 shares 51% sequence identity with the PobA enzyme from *P. fluorescens* strain Pf-5 [21]. In addition, protocatechuate has been described as a central route for 3-HBA degradation by a 3-HBA 4-hydroxylase encoded by the *mobA* gene in bacteria. The *mobA* gene was not found in the LB400 genome.

### Growth of Strain LB400 on Gentisate, Protocatechuate and HBAs

**Figure 6:**
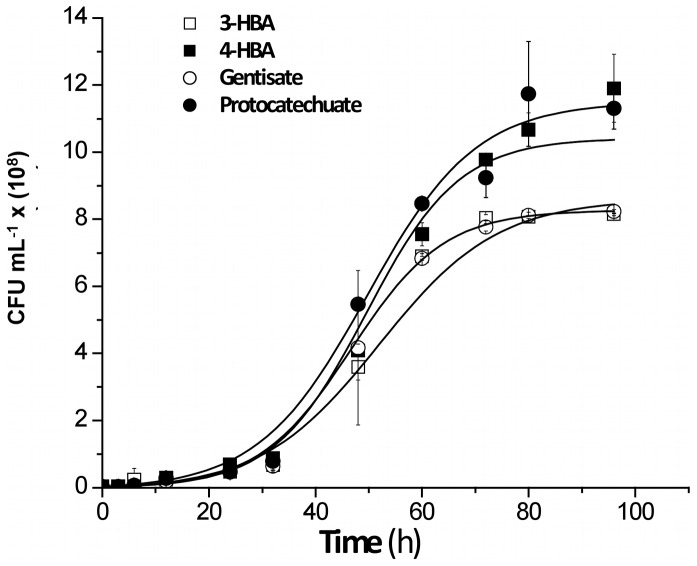
Growth of *B. xenovorans* LB400 on hydroxybenzoates, gentisate and protocatechuate. LB400 cells were grown in M9 minimal medium using gentisate (5 mM), protocatechuate (5 mM), 3-HBA (10 mM) or 4-HBA (5 mM) as sole carbon and energy source. Each point is an average ± SDs of results from, at least, three independent assays.

Sequence analyses of *B. xenovorans* LB400 genome revealed the presence of genes encoding the gentisate and the protocatechuate ring-cleavage pathways and 3-HBA and 4-HBA peripheral pathways. To investigate whether these catabolic pathways are functional, in a first approach growth assays were performed. The growth of *B. xenovorans* strain LB400 on the central metabolites gentisate and protocatechuate was studied. *B. xenovorans* LB400 was able to grow on gentisate (5 mM) and on protocatechuate (5 mM) as sole carbon and energy source (Fig. 6). In addition, the growth of *B. xenovorans* strain LB400 on 3-HBA or 4-HBA was studied. Strain LB400 was able to grow on 3-HBA (10 mM) and 4-HBA (5 mM) (Fig. 6). *B. xenovorans* LB400 attained higher cell concentration at stationary phase on protocatechuate (1.1 ×10^9^ CFU mL^−1^) and 4-HBA (1.0×10^9 ^CFU mL^−1^). The growth of strain LB400 on gentisate or 3-HBA yielded 7.0×10^8^ CFU mL^−1^ (Fig. 6). These results indicate functional 3-HBA and 4-HBA peripheral pathways and gentisate and protocatechuate central pathways in *B. xenovorans* LB400.

### Transcriptional Analyses of the *mhb* and *pca* Genes

**Figure 7:**
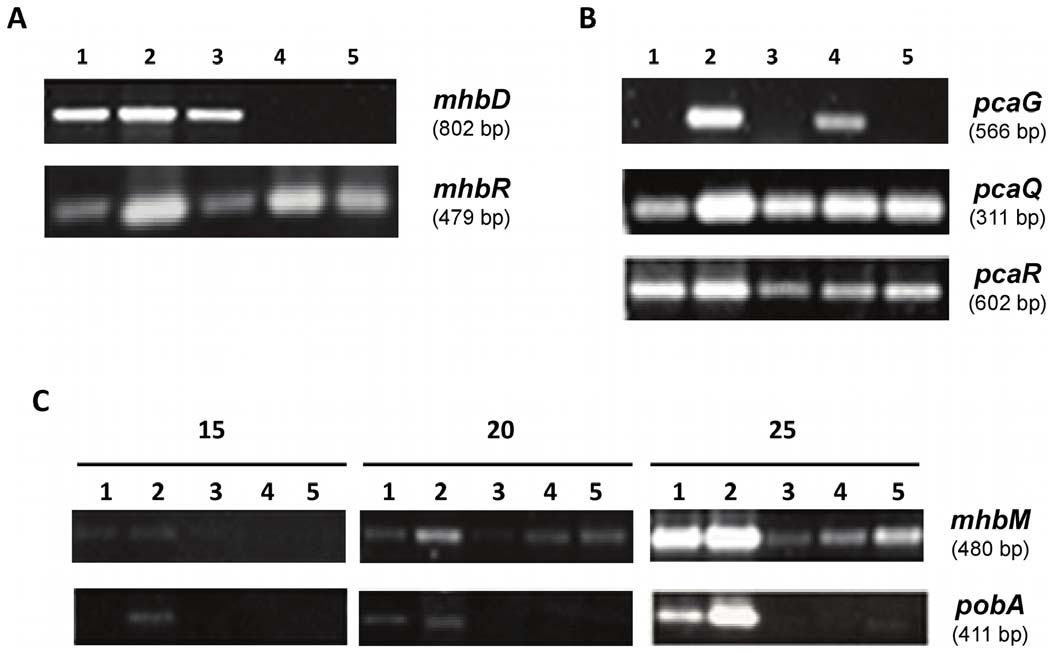
Transcriptional analysis of the *mhb* and *pca* genes during LB400 growth on hydroxybenzoates, gentisate and protocatechuate. LB400 cells were grown on 3-HBA (lane 1), 4-HBA (lane 2), gentisate (lane 3), protocatechuate (lane 4) and glucose (lane 5) as sole carbon source. (A) Transcription of *mhbD* and *mhbR* genes; (B) Transcription of *pcaG, pcaQ* and *pcaR* genes. (C) Transcription of *mhbM* and *pobA* genes. RT-PCR (15, 20 and 25 amplification cycles) assays were performed using RNA extracted from LB400 cells collected at mid exponential growth phase. Transcription of 16S rRNA gene was used as control (not shown).

In order to determine the role of predicted genes encoding the gentisate and protocatechuate ring-cleavage routes and 3-HBA and 4-HBA peripheral pathways in strain LB400, transcriptional analyses of key catabolic genes were performed. In addition, the expression of genes encoding potential transcriptional regulators was analyzed. The expression of the *mhbD* gene encoding the enzyme gentisate 1,2-dioxygenase during exponential growth phase of LB400 cells grown on gentisate, protocatechuate, 3-HBA, 4-HBA, and glucose was analyzed. The *mhbD* gene was transcribed during growth of strain LB400 in gentisate, 3-HBA and 4-HBA (Fig. 7). In contrast, expression of the *mhbD* gene was not observed during growth on protocatechuate or glucose (Fig. 7). The *mhbR* gene encoding a MarR-type transcriptional regulator was transcribed during LB400 growth on 3-HBA, 4-HBA, gentisate, protocatechuate and glucose (Fig. 7). These results suggest that gentisate, 3-HBA and 4-HBA or its metabolites induce transcription of the *mhbD* gene in *B. xenovorans* LB400. On the other side, the *mhbR* gene has probably a basal constitutive expression. Transcription analysis of the *mhbM* gene encoding the 3-HBA 6-hydroxylase was performed. The *mhbM* gene of strain LB400 was expressed was during growth on 3-HBA, 4-HBA, gentisate, protocatechuate and glucose (Fig. 7). However, higher expression of the *mhbM* gene on HBAs than on glucose was observed.

Transcription of the *pcaG* gene encoding the key protocatechuate 3,4-dioxygenase alpha subunit during growth of strain LB400 with different aromatic substrates was studied. Predicted *pcaG* gene was expressed during growth of strain LB400 on protocatechuate and 4-HBA (Fig. 7). However, *pcaG* transcripts were not observed during growth of strain LB400 on gentisate, 3-HBA and glucose. These results suggest that by 4-HBA, protocatechuate or its metabolites induce the transcription of the *pcaG* gene. Both *pcaQ* and *pcaR* genes encoding the transcriptional regulators of the *pcaGH* genes and the *pcaIJBDC* gene cluster were transcribed during LB400 growth on HBAs, gentisate, protocatechuate and glucose (Fig. 7). Therefore, the *pcaQ* and *pcaR* genes have probably a basal constitutive expression.

Transcription of the *pobA* gene encoding the 4-HBA 3-monooxygenase of strain LB400 was analyzed. The *pobA* gene was expressed during LB400 growth only on 3-HBA, 4-HBA and glucose (Fig. 7). The *pobA* gene expression was higher during growth on HBAs than on glucose.

These results indicate that expression of the *mhb* and *pca* genes encoding catabolic enzymes is regulated in LB400 cells. These regulations are probably exerted by MarR-type and LysR-type transcriptional regulators. This study also suggests a regulated expression of the *mhbM* gene encoding the peripheral 3-HBA 6-hydroxylase and of the *pobA* gene encoding the peripheral 4-HBA 3-monooxygenase but with a basal constitutive expression of the *mhbM* gene. Overall, the transcriptional analyses suggest that 3-HBA is catabolized and funneled exclusively through the gentisate central pathway, whereas 4-HBA can be degraded through the protocatechuate and gentisate central pathways in strain LB400.

### Gentisate and Protocatechuate Ring-cleavage Activities

**Figure 8:**
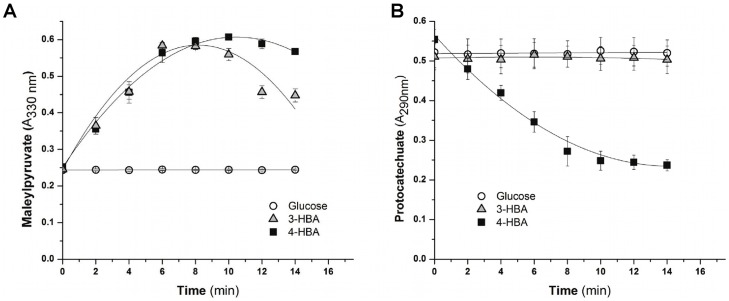
Gentisate and protocatechuate dioxygenase activities of *B. xenovorans* LB400. Cells were grown on 3-HBA (black square), 4-HBA (empty square) and glucose (empty circles) as sole carbon source. (A) Gentisate 1,2-dioxygenase activity measured by maleylpyruvate formation. (B) Protocatechuate 3,4-dioxygenase activity measured by protocatechuate disappearance. Each point is an average ± SDs of values from three independent assays.

To further characterize the gentisate and protocatechuate central pathways in *B. xenovorans* strain LB400, the activities of the key enzymes gentisate 1,2-dioxygenase and protocatechuate 3,4-dioxygenase were measured. Both dioxygenase activities were determined in crude extract of cells cultured until mid exponential phase in 3-HBA, 4-HBA and glucose. Higher gentisate 1,2-dioxygenase activity was observed in cells grown on 3-HBA (0.053±0.001 U mg^−1^ protein) and 4-HBA (0.049±0.004 U mg^−1^ protein), whereas the gentisate 1,2-dioxygenase activity was not detected in glucose-growth cells (Fig. 8A). LB400 cells grown on 4-HBA showed high protocatechuate 3,4-dioxygenase activity (0.15±0.02 U mg^−1^ protein), whereas the protocatechuate dioxygenase activity was not observed in LB400 cells grown on 3-HBA and glucose (Fig. 8B). Enzyme activity assays suggest an active protocatechuate pathway in 4-HBA-grown cells, whereas the gentisate central pathway was found to be active in both 3-HBA- and 4-HBA-grown cells.

## Discussion

This study has focused on a genomic analysis on the gentisate and protocatechuate catabolic pathways and its related peripheral pathways in *B. xenovorans* LB400. Experimental evidence was provided on the functionality of these ring-cleavage pathways, and their roles during 3-HBA and 4-HBA catabolism were proposed. Strain LB400 is able to grow on gentisate as sole carbon source, indicating a functional gentisate catabolic pathway. The gentisate catabolic pathway is present only in few *Burkholderia* strains [1]. Both *mhbRTDHI* gene clusters were found in two identical and adjacently located segments of 30 genes in LB400 major chromosome, indicating a gene cluster duplication process. Gene duplication is frequently observed in aromatic catabolic pathways and suggests functional redundancies, which could potentially increase the bacterial fitness and its adaptation to adverse environmental conditions. The *mhbDHI* gene cluster organization is similar in strains from *Burkholderiales* order including *B. xenovorans* LB400. However, the complete *mhbRTDHI* gene cluster with a coding sequence of unknown function between *mhbT* and *mhbD* was found in strain LB400, which has not been observed in other bacteria and its function remains to be elucidated.

Growth studies indicated that a 3-HBA peripheral pathway is present in *B. xenovorans* LB400. Gentisate has been described as the main ring-cleavage pathway for 3-HBA assimilation in bacteria, such as *K. pneumoniae* M5a1 [11], *Corynebacterium glutamicum* RES167 [44] and *Rhodococcus* sp. strain NCIMB 12038 [45]. In *B. xenovorans* strain LB400 the *mhbM* gene encoding the 3-HBA 6-monooxygenase is located at C2, whereas the *mhbRTDHI* gene cluster is located at C1 (Fig. 1). In contrast, in strains M5a1, RES167, NCIMB 12038 and in *Comamonas* and other *Burkholderia* strains, a 3-HBA 6 hydroxylase-encoding *mhbM* gene is usually found within the *mhbDHI* gene cluster. An increased transcription of *mhbM* gene in strain LB400 was observed during growth on 3-HBA. Therefore, we propose that the predicted *mhbM* gene product of strain LB400 is involved in the hydroxylation of 3-HBA to gentisate.

It is worth noting that *B. xenovorans* LB400 was able to grow using 10 mM 3-HBA, whereas no growth of LB400 cells was achieved on 5 mM 3-HBA. We propose that a 3-HBA (5 mM) did not support the growth of *B. xenovorans* strain LB400, probably due to an insufficient transport of 3-HBA across the membrane. These results suggest that strain LB400 lacks an active transport for 3-HBA. 4-HBA catabolism through the gentisate pathway has been described in bacterial strains from *Bacillus* genus [46] and the archaeal isolate *Haloarcula* sp. strain D1 [42]. *B. xenovorans* LB400 was able to grow using 4-HBA (5 mM) as sole carbon source. The LB400 growth on 4-HBA (5 mM) but not on 3-HBA (5 mM) suggests an efficient transport of 4-HBA rather than 3-HBA through the membrane by an active 4-HBA transport system. This is supported by our bioinformatics analyses that indicated that the *mhbT* gene product of strain LB400 has 33% sequence identity with the PcaK protein of *P. putida* PRS2000, which is an active transporter system for 4-HBA [29], [33]. The *mhbT* gene of *B. xenovorans* LB400 encodes a MFS transporter of the AAHS family that may be involved in the active transport of gentisate and 4-HBA.

**Figure 9:**
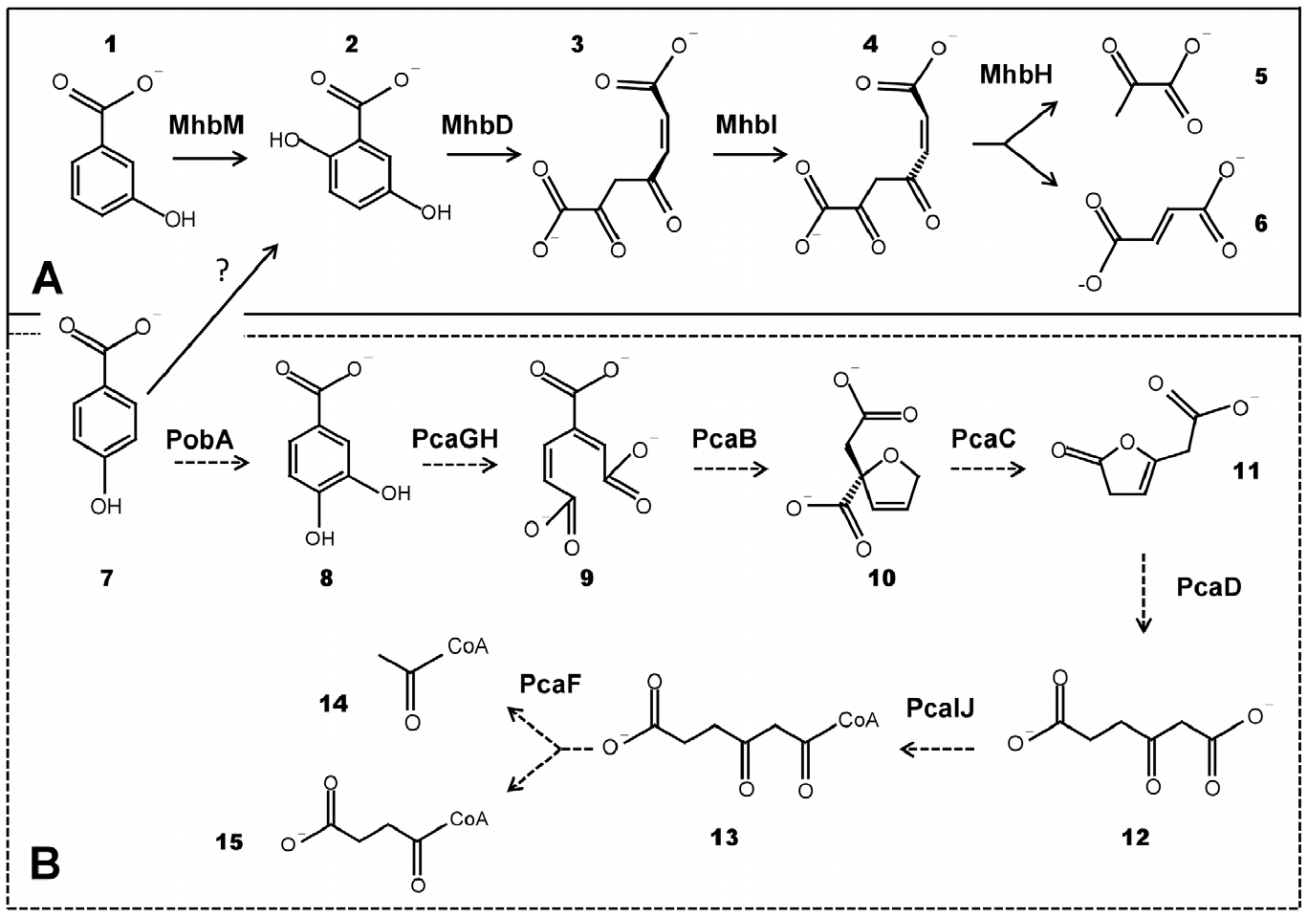
Models of 3-HBA and 4-HBA catabolic pathways in *B. xenovorans* LB400. (A) 3-HBA and 4-HBA catabolism via the gentisate central pathway (continuous line). The substrates and products are: 3-hydroxybenzoate (1); gentisate (2); maleylpyruvate (3); fumarylpyruvate (4); pyruvate (5); fumarate (6) and 4-hydroxybenzoate (7). The enzymes are MhbM (3-HBA 6-hydroxylase), MhbD (gentisate 1,2-dioxygenase), MhbI (maleylpyruvate isomerase) and MhbH (fumarylpyruvate hydrolase). The gene encoding the 4-HBA 1-hydroxylase is unknown. (B) 4-HBA catabolism via protocatechuate (dotted line). The substrates and products are: 4-hydroxybenzoate (7); protocatechuate (8); 3-carboxy-*cis,cis*,-muconate (9); 4-carboxymucolactone (10); β-ketoadipate enol-lactone (11); β-ketoadipate (12); β-ketoadipyl-CoA (13); Acetyl-CoA (14); Succinyl-CoA (15).The enzymes are: PobA (4-hydroxybenzoate 3-monooxygenase), PcaGH (protocatechuate 3,4-dioxygenase), PcaB (3-carboxymuconate cycloisomerase), PcaC (4-carboxymuconolactone decarboxylase), PcaD (β-ketoadipate enol-lactone hydrolase), PcaJI (β-ketoadipate:succinyl-CoA tranferase) and PcaF (β-ketoadipyl-CoA thiolase).

Genomic analyses suggest that the *mhbR* gene, which is upstream and divergently transcribed to the *mhbT* gene, encodes for a transcriptional regulator of the *mhbT* gene in strain LB400. The *mhbR* gene of strain LB400 was transcribed during growth on 3-HBA, 4-HBA, gentisate, protocatechuate and glucose, suggesting a basal constitutive expression. The *mhbR* gene product from *B. xenovorans* LB400 shares 30% sequence identity with MarR-type transcriptional regulators from the *mhb* gene cluster from *P. naphthalenivorans* CJ2 [28] and *C. testosteroni* CNB-2 [47]. MarR regulators are winged helix-turn-helix DNA-binding proteins that respond to specific ligands and act as dimmers. In absence of the ligand, MarR regulators bind to palindromic sequences within the promoter, generally causing transcriptional repression [40]. We propose that the MarR-type regulator encoded by the *mhbR* gene from *B. xenovorans* LB400 is a transcriptional repressor that regulates the expression of the *mhbT* gene. Transcriptional analysis showed that the *mhbD* gene encoding a gentisate 1,2-dioxygenase was expressed during growth on gentisate, 3-HBA and 4-HBA, but not on glucose. Transcriptional analyses suggest that either 3-HBA, 4-HBA, gentisate or its metabolic intermediates induced the transcription of the *mhb* gene cluster. The expression of *mhbD* gene correlates with an increased gentisate 1,2-dioxygenase activity observed in LB400 cells grown on 3-HBA and 4-HBA. Genomics analysis showed the presence of a conserved sequence (5′-T-N_11_-A-3′) critical for LysR-type transcriptional regulator binding, located upstream of the -35 box of BxeA2626 and BxeA4527 genes. However, a LysR-type transcriptional regulator gene was not found in the neighborhoods of both *mhb* gene clusters. Although further studies are needed to survey the regulation mechanisms of 3-HBA and 4-HBA degradation pathways via gentisate in strain LB400, these genomic analyses suggest that *mhbDHI* cluster is regulated by a LysR-type transcriptional regulator in strain LB400. Overall, transcriptomic analyses and dioxygenase activity assays indicate that the gentisate pathway in strain LB400 is active during 3-HBA and 4-HBA degradation, suggesting that both substrates may be funneled into the gentisate central pathway (Fig. 9). We propose that a 4-HBA 1-hydroxylase could be involved in the conversion of 4-HBA into gentisate in *B. xenovorans* LB400. Experimental data with deuterated substrates in the archaeal strain *Haloarcula* sp. D1 supported the mechanism in which 4-HBA is converted into gentisate by hydroxylation of 4-HBA at C-1, with a concomitant 1,2-carboxyl group migration. The transformation is called NIH shift and is catalysed by a 4-HBA 1-hydroxylase [42]. Although no gene sequence information of the 4-HBA 1-hydroxylase enzyme is available to perform a genomic search, an active gentisate pathway during 4-HBA assimilation suggests a 4-HBA 1-hydroxylase activity in *B. xenovorans* strain LB400. Recently, we reported that *B. xenovorans* strain LB400 degrades 4-hydroxyphenylacetate via homogentisate, in which also a migration of the acetate group by a NIH shift reaction may also be involved [4]. Hydroxylation of monosubstituted aromatic rings at C-1, followed by a migration of the carboxymethyl side chain to C-2 has been described in *P. acidovorans* and *Nitrosomonas europaea* 19718 [48], [49]. Future studies are needed to characterize the genes encoding the 4-HBA 1-hydroxylase that converts 4-HBA into gentisate in bacteria, including *B. xenovorans* strain LB400.

Most bacteria metabolize 4-HBA by hydroxylation at C-3 to yield protocatechuate as central intermediate [1], [2]. The 4-HBA 3-monooxygenase encoded by the *pobA* gene catalyzes 4-HBA conversion into protocatechuate in *Pseudomonas* strains [21], [22]. Genomic analyses revealed the presence of the *pca* genes encoding the complete protocatechuate central pathway are located at C2 and *pca* gene copies are located at C1 and MP, while the *pobA* gene is separately located at C1 in strain LB400 genome. Spreading of *pca* genes and genes encoding its related peripheral reactions is also observed in *Betaproteobacteria*, such as *Cupriavidus* (Fig. 4), *Burkholderia* and *Ralstonia* strains [1]. Diverse *pca* gene organizations have been reported in bacteria [1], [2], [20]. The *pcaGH* genes are clustered with other *pca* genes of the protocatechuate route and related peripheral pathways in the *Gammaproteobacteria A. baylyi* ADP1 and *P. putida* KT2440, the *Alphaproteobacteria* strains *Agrobacterium tumefaciens* C58 and *Sinorhizobium meliloti* 1021, and the *Betaproteobacteria* from the *Cupriavidus* genus such as *C. metallidurans* CH34, *C. pinatubonensis* JMP143 and *C. necator* H16 [1]. In contrast, in *B. xenovorans* LB400 and other bacterial strains from the *Burkholderia* genus, the *pcaGH* genes are not clustered with other *pca* genes. *B. xenovorans* strain LB400 possesses the *pcaQHG* and the *pcaRIJBDC* gene clusters and separately *pcaF* gene copies. In addition, LB400 genome contains a *pcaIJ* gene cluster and *pcaB, pcaD and pcaJ* gene single-copies, indicating genetic redundancy.

*B. xenovorans* LB400 was able to grow on 4-HBA and protocatechuate, indicating functional 4-HBA peripheral and protocatechuate ring-cleavage pathways. Transcriptional analyses showed that *pcaG* gene encoding the protocatechuate 3,4-dioxygenase alpha subunit was expressed during protocatechuate and 4-HBA degradation by strain LB400. The expression of the *pcaG* gene during 4-HBA degradation correlates with an increased protocatechuate 3,4-dioxygenase activity observed in LB400 cells extracts grown on 4-HBA. In contrast, neither *pcaG* gene expression nor protocatechuate 3,4-dioxygenase activity was observed in 3-HBA and glucose-grown LB400 cells. These results suggest that 4-HBA is exclusively degraded via protocatechuate, in which a hydroxylation of 4-HBA at C-3 occurred for protocatechuate formation. The *pobA* gene of strain LB400 was transcribed during growth on 4-HBA, suggesting that the *pobA* gene product is involved in 4-HBA hydroxylation to produce protocatechuate in *B. xenovorans* strain LB400 (Fig. 9). *B. xenovorans* LB400 as well as most strains belonging to *Burkholderia* and *Ralstonia* genera that possess the *pcaGH* genes have a 4-HBA 3-hydroxylase-encoding gene, indicating that protocatechuate is the preferred catabolic pathway for 4-HBA degradation. In addition, these results suggest that either 4-HBA, protocatechuate or its metabolic intermediates induced the transcription of protocatechuate 3,4-dioxygenase-encoding genes. The *pcaQ* gene adjacently located to *pcaGH* genes encodes a LysR-type transcriptional regulator and was transcribed during growth on 4-HBA, protocatechuate, 3-HBA, gentisate and glucose, suggesting a constitutive expression of the *pcaQ* gene. Thus, the PcaQ LysR-type transcriptional regulator of strain LB400 probably controls the expression of the *pcaGH* genes during 4-HBA and protocatechuate degradation in strain LB400. LysR-type transcriptional regulators act as activators during aromatic compounds catabolism. In general, the inducers are metabolic intermediates of the catabolic pathway [2], [20]. The PcaQ regulator from strain LB400 probably acts as a transcriptional activator of the expression of the divergently transcribed *pcaGH* genes, like other PcaQ proteins that are transcriptional activators of the *pca* genes in *Agrobacterium tumefaciens* and *Sinorhizobium meliloti* [32], [43]. The *pcaR* gene adjacently located upstream of the *pcaIJBDC* gene cluster encodes a LysR-type transcriptional regulator and was transcribed during growth on 4-HBA, protocatechuate, 3-HBA, gentisate and glucose. In *P. putida* strains PRS2000 and KT2440, IclR-type transcriptional regulators regulate the *pca* gene cluster encoding enzymes for the degradation of 3-carboxy-*cis*,*cis*-muconate into Krebs cycle intermediates [2], [20]. We propose that the PcaR protein may regulate the expression of the *pcaIJBDC* gene cluster in strain LB400. The *pcaRIJBDC* gene organization with a LysR-type regulator with high sequence identity (95%) was observed in the closely relative strain *B. phytofirmans* PsJN. In accordance with this, it has been reported that all strains of the ‘*B. cepacia* complex’ as well as *B. phymatum* STM815 and *B. glumae* BGR1 possess a LysR-type transcriptional regulator gene clustered with the *pcaIJBDC* genes [1]. It is likely that the *pcaR* gene product of strain LB400 acts as a transcriptional activator of the expression of the divergently transcribed *pcaIJBDC* gene cluster. Further studies are required to confirm that PcaQ and PcaR proteins regulate the expression of the *pcaHG* and *pcaIJBDC* gene clusters in *B. xenovorans* LB400. The protocatechuate pathway is widespread among pathogenic and environmental bacteria, and only a small number of strains lack this pathway. Therefore, the protocatechuate catabolic pathway can be an important catabolic feature and play a key role to improve bacterial fitness in the environment and in pathogenesis.

In this report, we described functional gentisate and protocatechuate ring-cleavage pathways in *B. xenovorans* LB400, revealing inducible expression of key genes encoding gentisate and protocatechuate dioxygenases. Additionally, in this study 3-HBA and 4-HBA peripheral reactions in strain LB400 were reported. Based on genomic analyses, growth assays, gene expression analyses and key dioxygenase activities, we propose that 3-HBA degradation is channeled exclusively through the gentisate central pathway in *B. xenovorans* strain LB400, whereas 4-HBA can be degraded and funneled actively into the protocatechuate and gentisate central pathways (Fig. 9). This report reveals novel catabolic capabilities of *B. xenovorans* LB400, which can use two different ring-cleavage pathways for the metabolism of 3-HBA and 4-HBA. This study confirms that *B. xenovorans* LB400 possesses a high metabolic versatility, which is reflected on its extensive gene and functional redundancy for the degradation of aromatic compounds.
